# Predicting Gains With Visuospatial Training After Stroke Using an EEG Measure of Frontoparietal Circuit Function

**DOI:** 10.3389/fneur.2018.00597

**Published:** 2018-07-24

**Authors:** Robert J. Zhou, Hossein M. Hondori, Maryam Khademi, Jessica M. Cassidy, Katherine M. Wu, Derek Z. Yang, Nikhita Kathuria, Fareshte R. Erani, Lucy Dodakian, Alison McKenzie, Cristina V. Lopes, Walt Scacchi, Ramesh Srinivasan, Steven C. Cramer

**Affiliations:** ^1^Department of Neurology, University of California, Irvine, Irvine, CA, United States; ^2^Department of Informatics, University of California, Irvine, Irvine, CA, United States; ^3^Department of Physical Therapy, Chapman University, Irvine, CA, United States; ^4^Institute for Software Research, University of California, Irvine, Irvine, CA, United States; ^5^Department of Cognitive Sciences, University of California, Irvine, Irvine, CA, United States; ^6^Department of Biomedical Engineering, University of California, Irvine, Irvine, CA, United States; ^7^Department of Anatomy & Neurobiology, University of California, Irvine, Irvine, CA, United States; ^8^Department of Physical Medicine & Rehabilitation, University of California, Irvine, Irvine, CA, United States

**Keywords:** stroke, rehabilitation, electroencephalography, augmented reality, parietal lobe, motor, therapy, coherence

## Abstract

The heterogeneity of stroke prompts the need for predictors of individual treatment response to rehabilitation therapies. We previously studied healthy subjects with EEG and identified a frontoparietal circuit in which activity predicted training-related gains in visuomotor tracking. Here we asked whether activity in this same frontoparietal circuit also predicts training-related gains in visuomotor tracking in patients with chronic hemiparetic stroke. Subjects (*n* = 12) underwent dense-array EEG recording at rest, then received 8 sessions of visuomotor tracking training delivered via home-based telehealth methods. Subjects showed significant training-related gains in the primary behavioral endpoint, Success Rate score on a standardized test of visuomotor tracking, increasing an average of 24.2 ± 21.9% (*p* = 0.003). Activity in the circuit of interest, measured as coherence (20–30 Hz) between leads overlying ipsilesional frontal (motor cortex) and parietal lobe, significantly predicted training-related gains in visuomotor tracking change, measured as change in Success Rate score (*r* = 0.61, *p* = 0.037), supporting the main study hypothesis. Results were specific to the hypothesized ipsilesional motor-parietal circuit, as coherence within other circuits did not predict training-related gains. Analyses were repeated after removing the four subjects with injury to motor or parietal areas; this increased the strength of the association between activity in the circuit of interest and training-related gains. The current study found that (1) Eight sessions of training can significantly improve performance on a visuomotor task in patients with chronic stroke, (2) this improvement can be realized using home-based telehealth methods, (3) an EEG-based measure of frontoparietal circuit function predicts training-related behavioral gains arising from that circuit, as hypothesized and with specificity, and (4) incorporating measures of both neural function and neural injury improves prediction of stroke rehabilitation therapy effects.

## Introduction

Stroke remains a leading cause of adult disability. A number of treatment modalities are under study to improve outcomes, particularly for arm motor deficits, which are present in >80% of patients with stroke (1). These efforts are complicated by the fact that stroke is a heterogeneous condition, and so restorative therapies are not likely to benefit from a one-size-fits-all approach. Therefore, an intense area of research is the evaluation of methods to identify the target population for post-stroke restorative therapies.

One approach to identifying predictors of treatment response emphasizes measuring brain function at the circuit level. Measures of functional connectivity reliably correspond to behavioral deficits (2–5) and after stroke such measures can predict spontaneous (6) and treatment-related recovery (7–10). Furthermore, combining anatomical measures of injury with functional connectivity measurement improves the predictive value compared to either alone (9–12).

The current study extended this approach by providing training that targeted a specific neural circuit in order to test whether baseline function of a specific frontoparietal circuit predicts training-related gains in a behavior arising from that circuit's function. The motor-parietal circuit targeted in the current study was identified in a prior study of healthy subjects (13) that identified predictors of training-related gains in visuomotor tracking skill, a behavior central to many forms of rehabilitation therapy after stroke. In that study, a measure of coherence in the high beta band (20–30 Hz), recorded at rest using dense-array EEG, between leads overlying left primary motor area (M1) and a left parietal area (PAR) was a strong predictor of motor skill acquisition, exceeding the information provided by baseline behavior and demographic data.

*The primary hypothesis* of the current study was that high beta coherence in the same frontoparietal circuit, measured using identical EEG acquisition and analysis methods as in the prior study (13), would again predict training-related gains in visuomotor tracking skill. *Coherence is of interest* because it is considered to be a measure of functional connectivity between two brain regions. Coherence is estimated from EEG electrodes overlying the corresponding regions (14). Coherence ranges from zero to one, with a coherence value near one indicating EEG signals have similar phase and amplitude difference at all time points, and a coherence value near zero indicating signals have a random difference in phase and amplitude. Although coherence has been widely adopted in EEG studies as a surrogate marker of communication between cortical neural sources (14), there is potential that an observed increased in coherence may result from increased input from a tertiary common neural source (14, 15). Changes in coherence after stroke are thus seen as changes in functional connectivity and might result from a combination of injury (to cortical EEG sources or to white matter tracts connecting them) or from functional changes—a distinction examined in the current report. *The frontoparietal circuit is of interest* because of its established importance to visuomotor tracking, a behavior that is important to many activities of daily living and rehabilitation therapy regimens. Parietal cortex has direct and indirect anatomical connections with the precentral gyrus (16–19), which transmit information form parietal operations that include locating of the arm in space in a body-centered coordinate system (16, 20), processing spatial components of movement (21, 22), and transforming sensory information into information appropriate for action and thus providing visuomotor transformations (23) in relation to the dorsal visual stream (24) and in support of visuomotor tracking (25). The specific EEG leads defining the specific parietal-motor circuit of interest were defined in our prior study (13). *The high beta frequency range (20–30 Hz) is of interest* because it was informative in the prior study (13), and because it is known to be the frequency band most closely associated with function of the human motor system (26–28). The current study is focused on patients with stroke, among whom it is known that injury effects can be associated with changes in the distribution and magnitude of beta coherence (29–32).

The population evaluated in the current study was patients with chronic hemiparetic stroke. Training consisted of a 5-day protocol focused on visuomotor tracking, and was provided using augmented reality games, an approach we have found feasible in stroke survivors (33, 34). Three secondary hypotheses were (a) that training results would generalize but only to other visuomotor tracking assessments; (b) that incorporating a measure of anatomical injury would improve the relationship between circuit function and behavior; and (c) that only the hypothesized frontoparietal circuit would predict training-related gains, i.e., function of other circuits would not predict gains and thus current predictor findings would have specificity.

## Methods

### Study overview

Subjects meeting entry criteria (see Table 1) were recruited from the community via advertisements. Those meeting entry criteria underwent a 5-day protocol consisting of (a) testing at baseline, (b) training on visuomotor tracking skill across eight sessions, and (c) testing post-training Testing consisted of visuomotor tracking assessment plus a resting EEG recording. The first training session occurred at the lab in UC Irvine following baseline assessments. The second training session occurred later that same day (Day 1), and the remaining six training sessions were twice/day over the next 3 days (Days 2–4) and took place in the subject's home. Subjects returned to the lab on Day 5 for post-training assessments.

**Table 1:** Entry and exclusion criteria.

**Entry Criteria**	**Exclusion Criteria**
Age > 18 years	Significant difficulty maintaining attention or understanding instructions
Prior diagnosis of stroke, radiologically confirmed	Advanced liver, kidney, heart, or lung disease
English speaking	Major neurological, psychiatric, or medical disease
Arm weakness arising from stroke	Co-existing diagnosis having a major effect on arm/hand function
Able to attend and participate in all visits and sessions	Unable to successfully perform the test exercise examples
Ability to move at least 3 blocks over 60 s on the Box & Block test using the paretic arm	

*The primary behavioral endpoint* of this study was the ability to successfully track a moving target, calculated as the Success Rate (SR) score and expressed as percent change over time (i.e., from pre-training to post-training). The secondary behavioral endpoint was the percentage of error a patient made tracking the moving target, referred to as Error Rate (ER) score.

This study was carried out in accordance with the recommendations of, and the protocol was approved by, the University of California, Irvine Institutional Review Board. All subjects gave written informed consent in accordance with the Declaration of Helsinki.

### Subject characteristics

**Figure 1:**
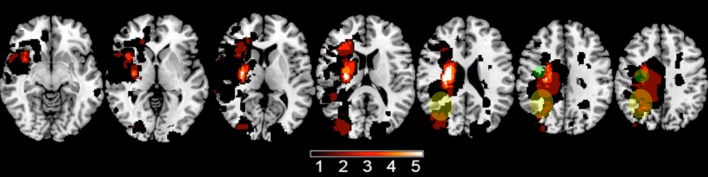
A lesion overlay plot shows the 12 infarcts among study subjects. The color bar indicates the number of subjects with an infarct at any given brain pixel. The green circles approximate the location of the iM1 region analyzed, and the yellow ellipses approximate the location of the iPAR region.

A total of 12 subjects were studied (Table 2). There was 1 female and 11 males. All were right-handed. The infarct affected the left brain in seven and right brain in five. No subject was receiving concurrent occupational or physical therapy. Average years of education was 16.1 ± 3.1. The stroke was ischemic in 10 and hemorrhagic in two. The infarct (Figure 1) injured the hand area of primary motor cortex in four subjects, among whom 9.4 ± 10.0% of this region was injured. The infarct injured the parietal lobe in three of these subjects, among whom superior parietal lobule injury averaged 27.1 ± 26.3% and inferior parietal lobule injury averaged 13.3 ± 10.4%. Subjects had mild to moderate motor impairment at baseline, e.g., median Box and Blocks score was 13 [IQR = 7–20], which was 18.2% [IQR 8.6–27.8] of age/gender-adjusted normal values (35).

**Table 2:** Subject characteristics.

**Baseline (Pre-Training)**		**Post-Training scores**	**Percent change over one week**	
			**Value**	***P***
Age	63.8 ± 10.7			
Time post stroke (months)	35 ± 26			
Infarct volume (cc)	17.2 ± 25.3			
Geriatric depression score	2.4 ± 2.2			
Box & blocks score	14.9 ± 11.8			
Symbol digit modality test (out of 110)	34.3 ± 10.7	36.2 ± 13.8	12.7 ± 13.0	0.004
Bells test (Total number circled out of 35)	31.1 ± 6.3	34 ± 1.8	14.8 ± 29.4	0.039
Bells test (Time in seconds)	203.5 ± 54.4	172.6 ± 71.8	−11.2 ± 37.4	0.37
Benton judgment of line orientation (out of 30)	24.8 ± 4.8	25.3 ± 5.4	1.6 ± 9.4	0.57
Trailmaking A (Time in seconds)	49.6 ± 30.1	48.5 ± 34.3	−4.5 ± 15.4	0.38
Trailmaking B (Time in seconds)	107.7 ± 67.8	97.6 ± 74.3	−11.2 ± 27.8	0.25
Success rate score	60.5 ± 11.5	74.0 ± 13.2	24.2 ± 21.9	0.003
Error rate score	32.1 ± 5.3	26.5 ± 5.8	16.7 ± 16.9	0.01

*All subjects completed all 25 Trailmaking A and B targets. Values are mean ± SD. P refers to significance of change over 1 week*.

The AR system set up in each subject's home operated correctly throughout the week. All but one subject completed all eight visuomotor training sessions. One subject skipped a single home-based session due to a schedule conflict. Although subjects were instructed to perform only two training sessions each day, one subject performed an extra three sessions during the week.

### Behavioral assessments

At the baseline visit (Day 1), prior to any visuomotor skill testing or training, demographic data were recorded, as was handedness (36). Depression was scored using the Geriatric Depression Scale (37), which ranges from 0 to 15, with higher scores indicating greater depression and with a score of 5 or higher suggesting depression. Manual dexterity was measured using the Box & Blocks Test (38), which counts the number of blocks transferred across a table and over an obstacle by the paretic arm during 60 s.

Subjects also underwent serial behavioral testing, once at the baseline visit (Day 1) and again post-training (Day 5); for any given subject, a single examiner performed all assessments. Next, subjects were scored on three tests of visuospatial skill: (1) *The Symbol Digit Modalities Test*, which assesses divided attention, visual scanning, tracking, and motor speed (39). Subjects are given a score sheet in which they match symbols to corresponding digits. Subjects are given a 10-item practice before beginning the actual test. The test consists of 110 symbols and subjects are given 90 s to complete as many as possible, in sequential order. (2) *The Bells Test* (40), which assesses visual inattention. Subjects view a score sheet with 35 bells hidden among 14 possible distracter items. Subjects are instructed to circle as many bells on the scoring sheet as possible, with no time limit. The primary measure for the Bells Test is the number of bells circled; time to completion is also measured secondarily. (3) *The Benton Judgment of Line Orientation Short Form* (41), which assesses spatial perception and orientation. Subjects are presented with 30 items and for each compare the orientation of two lines displayed in a booklet to a fan of all possible line orientations. Both lines have to be correctly identified to be considered a correct response. Items were ordered based on difficulty and subjects would move up in difficulty to establish a basal rate of 6 correct items plus a ceiling rule of 6 incorrect items. As a broad test of cognitive status, subjects also completed the Trailmaking A & B tests (42); for Trailmaking A, subjects were given 2 min to complete the test, and for Trailmaking B subjects were given 4 min. Computerized testing of reaction time (mean of 20 runs) was also obtained.

### EEG studies

#### EEG acquisition

Dense-array EEG was acquired at two time points. The first was at the Day 1 baseline visit and was used to address the primary study hypothesis. The second was at the Day 5 post-training visit and was used to examine a secondary hypothesis regarding change in EEG in relation to training-related behavioral gains. Awake, resting-state EEG was acquired for 180 s. Data were collected using a 256-lead Hydrocel net at 1,000 samples/s with a high input impedance Net Amp 300 amplifier and Net Station 4.5.3 software (ElectricalGeodesics, Inc., Eugene, OR). EEG signal was referenced to Cz during recording and re-referenced to the average of all leads for analysis. EEG signal was recorded without bandpass filters. During EEG acquisition, participants were instructed to hold still with the forearms resting on the anterior thigh and to direct their gaze at a fixation cross.

#### EEG analysis

EEG data were exported to Matlab (R2015a, MathWorks, Inc., Natick, MA) for preprocessing. The continuous EEG signal was low-pass filtered at 50 Hz, segmented into non-overlapping 1-s epochs, and detrended. Visual inspection and independent components analysis were used in combination to remove extra-brain artifacts from the EEG, as described previously (13). This included removing epochs contaminated by overt muscle activity, eye blinks, eye movements, and heart rhythms (43).

Next, EEG data underwent an Infomax ICA decomposition [EEGLAB (44)]. Components that only occurred in one channel or with high activity in 35–50 Hz frequency band, as typified by muscle artifact, were automatically rejected. Of the remaining components, amplitude topography, frequency spectra, and component time series were inspected to identify eye blinks, eye movements, and heart rhythms, and were removed.

#### Extraction of EEG coherence measures

*The primary EEG endpoint* was coherence between a seed region, consisting of leads overlying ipsilesional hand of primary motor cortex (iM1), and an ipsilesional parietal lobe region (iPAR), measured in the high beta (20–30 Hz) band. The iPAR region contained 21 leads over the lateral PAR area and was identified in a prior EEG study as the area for which high beta band coherence with M1 predicted learning during visuospatial skill training in healthy young subjects (13). The iM1 seed region included C3 and the six immediately surrounding electrodes. Secondary EEG measures were high beta coherence between iM1 and leads overlying either (a) contralesional PAR, (b) contralesional M1, (c) ipsilesional frontal/dorsal premotor cortex, (d) ipsilesional prefrontal cortex, (e) ipsilesional medial PAR, and (f) as a negative control ipsilesional primary visual cortex.

### Visuomotor tracking skill training

#### Augmented reality system

Subjects sat with their paretic forearm on a desk onto which moving virtual targets were presented, and as part of game play had to track the targets to earn points. Subjects were introduced to the system and had the first training sessions during the baseline visit, then the same system was delivered to the subject's home, where subjects underwent seven additional training sessions over 4 days.

**Figure 2:**
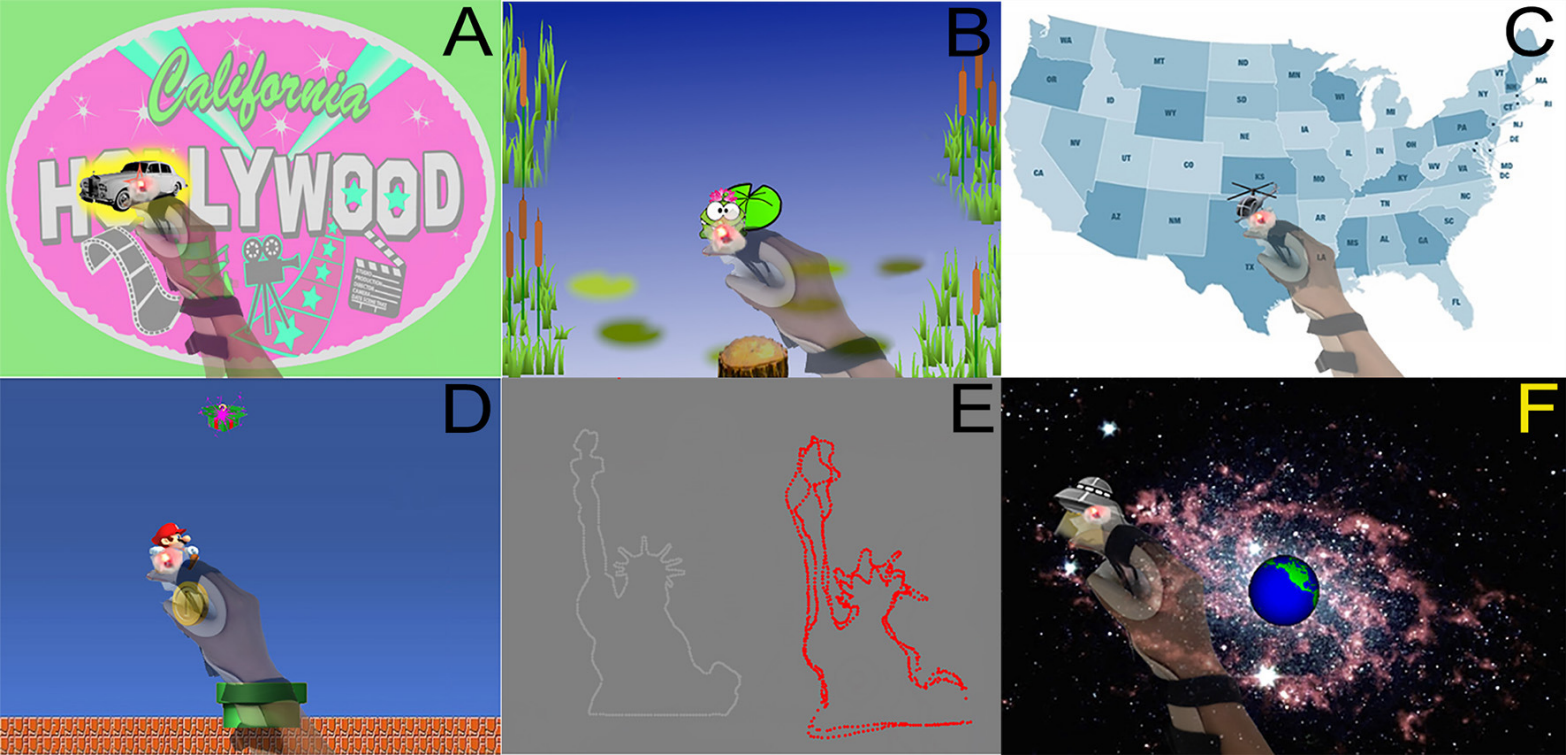
**(A)** Paparazzi game, whereby subject maintained the splint's red LED light over the white limousine. The yellow highlight around the car indicates that the subject is currently on the target. **(B)** Frog game, whereby a frog controlled by the subject's movements was to be kept on a lily pad. The lily pad turned bright green when the frog was on target. **(C)** Map game, during which the subject kept the LED over the flying helicopter as it traveled a circuitous route across the continental USA. **(D)** Mario game, whereby a Mario character controlled by the splint follows a gold coin and moves toward or away from a green gift box depending on the contents of the box when they are revealed. **(E)** Outline game, whereby a subject used the splint LED to carefully follow a target as it outlined 1 of 20 different shapes (such as the Statue of Liberty). After each round, the actual outlined shape (in white) was presented alongside the subject's attempts (in red). **(F)** UFO game, whereby the splint LED followed a UFO to prevent it from destroying the Earth.

The augmented reality (AR) approach used for visuomotor tracking skill training enabled subjects to interact in the real world tabletop workspace with virtual objects projected by a computer (45). The AR system consisted of a (1) computer (Dell Latitude E5420 laptop running Windows 7 Home Premium with Intel Core i5-2430M CPU @ 2.40 GHz and 4 GB RAM), (2) camera (PlayStation Eye), (3) projector (AAXA LED Android Pico Projector) that presented onto the tabletop images that were rapidly updated according to camera data, (4) chair, (5) table, and (6) LED-and infrared-equipped splint that the subject donned then moved around the tabletop. Before starting each session, subjects placed onto the paretic forearm a wrist splint that had two co-localized lights fixed on its superior aspect (Figure 2). One was an infrared (IR) light that allowed the camera to locate the splint's location on the tabletop, tracking hand movements in real time to drive game play, and the other was a red LED light that allowed the patient to see the precise spot that served as the cursor during game play. IR light data was fed to the computer, allowing the projector to provide tabletop game images that varied in real time according to the subject's game play movements. The projector was mounted 39″, and the camera 35″, above the table using an aluminum stand, generating a projected image size of 18 3/8″ × 29″ upon the table.

#### Baseline testing

On Day 1, each subject was tested to define the optimal speed for visuomotor tracking learning. The subject donned the splint and then played a tracking game for which the speed of the tracked target increased across successive rounds. The target moved in a cloverleaf pattern, and the subject was instructed to move their paretic hand as fast as possible while keeping the red LED over the target. Error was calculated as the Euclidean distance between the pixel representing the center of the splint's IR light (i) and the center of the target (j): [(x_i_-x_j_)^2^+(y_i_-y_j_)^2^]^0.5^. A distance <32 pixels was considered a success. The success rate for each round was calculated by dividing the total number of successful instances by the total number of instances. Across successive rounds, the speed of target movement was plotted against success rate, generating a sigmoidal curve for which the left tail asymptote was too simple/no error, and the right tail asymptote was too hard/maximum error. An individual subject's training speed was defined at 60% of the speed associated with maximum error.

Next, having defined the subject's training speed, a baseline test of visuomotor tracking skill was given, and repeated three times. The target moved in the same cloverleaf pattern as above, with each run of this test lasted 90 s.

The six games were then explained to the subject, including the target to be tracked in each game. The subject was taught how to independently don the wrist splint and how to initiate a training session on the computer. Next, the subject performed the first training session (see below) in the lab, under supervision. A member of the study team then delivered and setup the visuomotor tracking training system in the subject's home.

#### Eight training sessions spanning 4 days

The first training session was on Day 1, in the lab, following baseline testing. The remaining sessions were in the subject's home. The second training session was one on Day 1, at least 4 h after the first. Training sessions 3–8 were on Days 2–4, during which subjects were instructed to perform one training session in the morning and one in the evening with a minimum 4-h break between sessions. During each training session, subjects played each of the 6 games in succession. Training sessions lasted 20–30 min, depending on the subject's designated playing speed.

#### Repeat testing post-training

On Day 5, the subject returned to the lab. The same test of visuomotor tracking skill from Day 1 was again performed and again repeated three times, using the same individualized speed as at baseline. A member of the study team then removed the visuomotor tracking training system from the subject's home. Compliance with training at sessions was determined offline, once the system was returned to the lab.

#### Augmented reality games

For all six games (Figure 2), subjects were instructed to move the paretic hand to maintain the red LED over that game's target. Games were played at the subject's designated speed (see above). Each game lasted 3–5 min, with exact duration varying according to the subject's designated playing speed. All of these games were developed specifically for this experiment, and they contained enough instrumentation to collect all necessary data.

*(1) Paparazzi game*: Subjects were to maintain the splint's red LED atop a white limousine that drove around the table surface, and also stopped intermittently. The car glowed yellow when the subject was on target. When stopped, a celebrity photo was revealed and cheers were played if the subject was on target >60% of the time.

*(2) Frog game*: Subjects controlled the movements of a frog and were to keep the frog on a lily pad that moved across the tabletop. The lily pad turned bright green when the frog was on target. Intermittently a bubbling sound played, foreshadowing the appearance of a crocodile on the lily pad. Subjects were instructed to move away from the lily pad when the crocodile appeared.

*(3) Map game*: Subjects were to keep the LED atop a helicopter as it moved a circuitous route across a map of the continental USA. The helicopter stopped intermittently. If subject was able to stay on target >60% of the time prior to a stop, music played and images were displayed that were related to the city at which the helicopter was stopped.

*(4) Mario game*: Subjects controlled the movements of a Mario character to follow a gold coin as it moved around the bottom 2/3 of the projection. The top 1/3 of the projection had a moving green gift box. At various times, the gold coin disappeared as the green box opened to reveal either a sack of coins or a red monster. The subjects were instructed to move toward the sack of coins and away from the red monster.

*(5) Outline game*: Subjects maintained the splint LED atop a simple target displayed on the table that moved along a path that outlined a simple line drawing. Each round displayed 1 of 20 simple line drawings. Once the drawing was complete, the actual outlined shape was presented alongside the subject's attempt. Four shapes were outlined each round of game play.

*(6) UFO game*: Subjects maintained the splint LED atop a UFO as it flew around a background of space and the Earth. If subjects stayed on the UFO >60% of the time, the UFO exploded; if not, the Earth exploded.

#### Visuomotor tracking skill performance measures

The primary visuomotor tracking skill behavioral measure was the SR score, modeled after our prior approach to AR training (34). The SR score was determined by calculating the Euclidean distance between the pixel representing the center of the IR light and the center of the target. As during baseline testing, a distance <32 pixels was considered a success. This was repeated 30 times/s, and the final SR score for a given game was the proportion of assessments that were a success ^*^ 100. A secondary tracking skill behavioral measure was the ER score, which weighted each Euclidean distance by the magnitude of the distance.

### MRI data

Images demonstrating the index infarct were retrieved from medical records. Images available consisted of a clinical MRI in seven subjects (T2-weighted images and DWI) and a research MRI in five subjects (T1-weighted images), which in all cases were sufficient to visualize and outline the infarct (see Figure 1). Using methods described previously (46), a mask of each subject's stroke was generated by outlining the infarct in MRIcron. Masks were transformed into MNI stereotaxic space using FSL then binarized. Infarct volume was calculated, then the extent to which each infarct overlapped with two regions of interest [motor cortex (precentral gyrus) and parietal lobe (superior and inferior parietal lobules)] (47) was determined for each subject.

### Statistics

Bivariate analyses were used to determine correlation in ROI-based brain-behavior relationships. Statistical significance was set at *p* < 0.05. Nonparametric statistical analyses were employed because many measures were not normally distributed and could not be transformed to normality, thus analyses focused on correlation or prediction employed the Spearman rank correlation coefficient and analyses focused on within subject change over time employed the Wilcoxon signed rank test. A secondary analysis reanalyzed the primary study hypothesis excluding four subjects who had damage to either brain region (M1 or PAR lobe) from which the primary EEG outcome measure was derived. Statistical analyses were performed using JMP 8.0.2.

## Results

### Baseline visuomotor tracking performance

Baseline score for the primary endpoint, SR score, was 60.5 ± 11.5%. Baseline score for the secondary endpoint, ER score, was 32.1 ± 5.3%. Baseline SR and ER scores were related (*r* = −0.90, *p* < 0.001).

### Change in tracking performance across the week of visuomotor training

Subjects showed significant gains after training on the home-based AR system. Mean SR score increased (improved) to 74 ± 13.2% after training, a relative gain of 24.2 ± 21.9% (*p* = 0.003). Likewise, mean ER score fell (improved) to 26.5 ± 5.8% after training, a relative change of 16.7 ± 16.9% (*p* = 0.01). Change in SR score and in ER score were significantly related (*r* = 0.94, *p* < 0.0001).

Several other behavioral measures also showed significant change over time. Of the three visuospatial tasks tested before and after training, two showed significant improvement: Symbol Digit Modality score improved by 4.2 ± 3.8, a 12.7% relative improvement (*p* = 0.004), and the number of bells circled on the Bells Test rose by 2.9 ± 5.1, a 14.8% improvement (*p* = 0.039). Change in the score on the Benton Judgment of Line Orientation was 0.42 ± 2.1 (1.6% relative change) and was not significant (*p* = 0.57). Change over time in Trailmaking A time, Trailmaking B time, and reaction time was also not significant (*p* ≥ 0.25).

### Predicting change in visuomotor tracking performance

**Figure 3:**
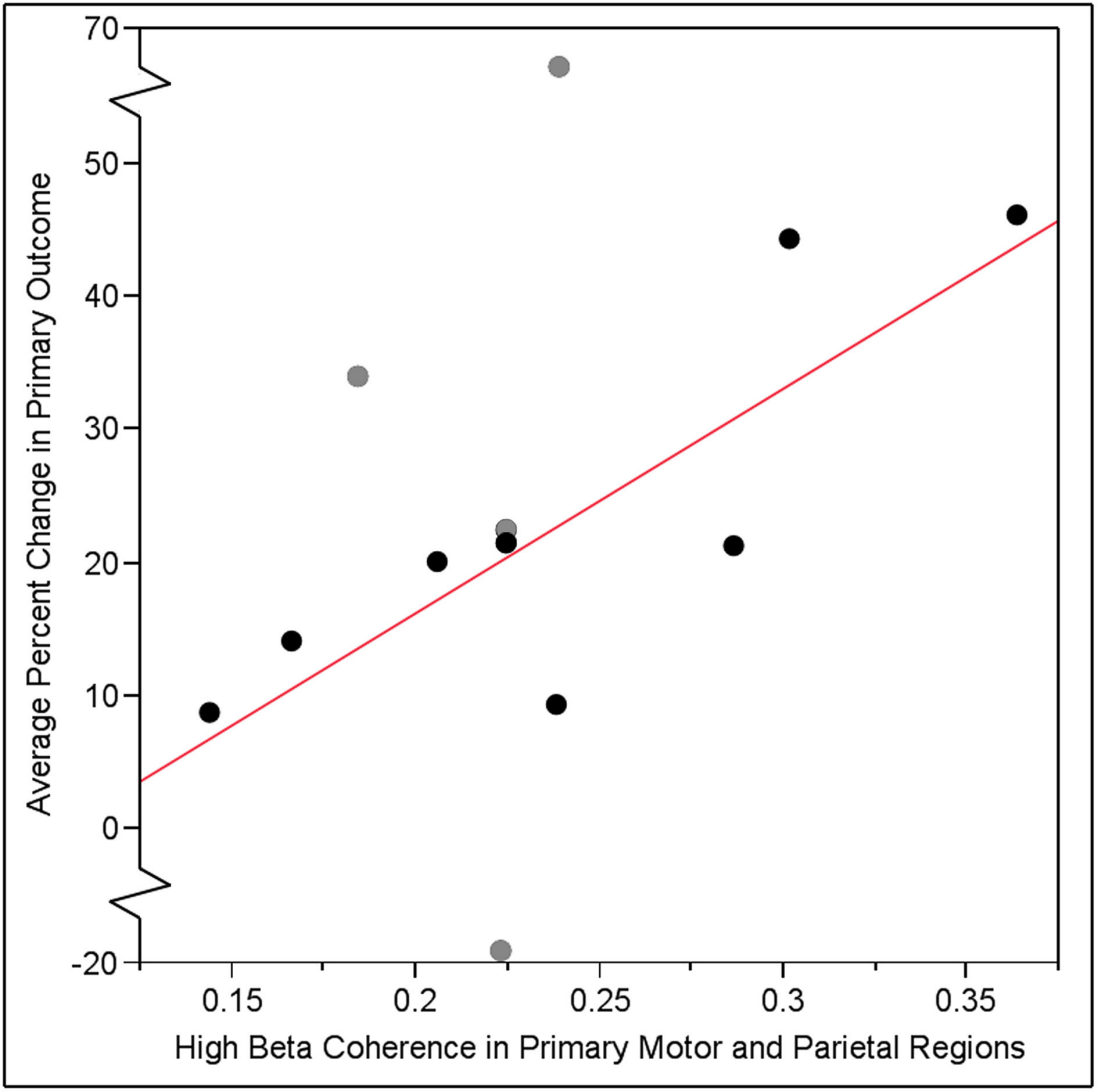
Training-related gain in visuomotor tracking, defined as the % change in SR score, pre- vs. post-training, increased linearly as a function of baseline coherence in the high beta band (20–30 Hz) between leads overlying ipsilesional primary motor cortex (iM1) and ipsilesional parietal lobe (iPAR) region identified by our group in a prior study (13) of visuomotor tracking training. The relationship between baseline EEG iM1-iPAR coherence and subsequent training-related gains was significant across all 12 subjects (*r* = 0.61, *p* = 0.037). When analysis was repeated excluding the four subjects (gray dots) who had injury to either iM1 or iPAR, this relationship was strengthened (*r* = 0.81, *p* = 0.015).

Data support the primary study hypothesis: individual gains in visuomotor tracking performance from baseline to post-training were significantly predicted by the hypothesized EEG-based measure of frontoparietal connectivity in the high beta band that was measured at baseline. Specifically, coherence between leads overlying iM1 and iPAR in the high beta band correlated with training-related gains in visuomotor tracking such that greater iM1-iPAR coherence at baseline predicted greater % change in the primary behavioral outcome measure, % change in SR score (*r* = 0.61, *p* = 0.037, Figure 3, black dots and gray dots). The same EEG measure showed a similar but non-significant relationship with the secondary behavioral outcome measure, % change in ER score (*r* = 0.52, *p* = 0.084). To further understand these relationships, we examined iM1-iPAR high beta coherence at baseline in relation to baseline, rather than training-related change in, tracking performance; baseline iM1-iPAR high beta coherence was not related to baseline SR score or baseline ER score (*p* > 0.9). We also examined whether change in high beta coherence between iM1-iPAR across the week of training correlated with change in tracking performance and it did not, neither for % change in SR score (*r* = −0.38, *p* = 0.23) nor % change in ER score (*r* = −0.40, *p* = 0.20).

To determine if injury to cortical regions underlying electrodes influences findings, we excluded four subjects with damage to either of the brain regions (iM1 or iPAR) in which coherence predicted training-related gains. When these four subjects were excluded, the strength of the relationship between baseline EEG iM1-iPAR high beta coherence and visuomotor tracking gains measured as change in SR score increased (*r* = 0.81, *p* = 0.015, Figure 3, black dots only). A similar strengthening of the relationship was found for the secondary endpoint, ER score, when excluding these four subjects but this did not reach significance (*r* = 0.62, *p* = 0.10).

As a control, we further hypothesized that prediction of training-induced gains in visuomotor tracking would be specific to connectivity in this ipsilesional frontoparietal circuit. To test this, we examined whether coherence in other circuits, both intra-hemispheric and inter-hemispheric, predicted the % increase in SR score, and none of these other circuits did (*p* > 0.1, Table 3).

**Table 3:** Baseline EEG prediction of training-related gain in visuomotor tracking.

**Coherence metric**	***r***	***p*-value**
iM1-iPAR	0.61	0.037
iM1-cPAR	0.20	0.53
iM1-cM1	0.36	0.25
iM1-iPMd	−0.06	0.86
iM1-iPf	0.36	0.25
iM1-iMedPr	0.49	0.11
iM1-iV1	−0.02	0.93

*Coherence was measured in the high beta band (20–30 Hz) and is reported for all 12 subjects. Training-related gain in visuomotor tracking is defined as the % change in SR score, pre- vs. post-training. Abbreviations indicate leads overlying: iM1, ipsilesional hand area of primary motor cortex; iPAR, ipsilesional parietal lobe region identified by our group in a prior study (13) of visuomotor tracking training; cPAR, contralesional parietal lobe region; cM1, contralesional primary motor cortex; iPMd, ipsilesional frontal/dorsal premotor cortex; Pf, ipsilesional prefrontal cortex; iMedPr, ipsilesional medial parietal area; iV1, ipsilesional primary visual cortex*.

As a further control, we explored the performance of coherence in the hypothesized iM1-iPAR circuit as a predictor but using other frequency bands besides the primary band of interest (high beta, 20–30 Hz). We found that M1-PAR coherence at baseline did not significantly predict % change in SR score in the theta (*r* = 0.11, *p* = 0.73), alpha (*r* = 0.13, *p* = 0.7), or low beta (*r* = 0.52, *p* = 0.087) frequency bands, but did predict % change in SR score in the delta band (*r* = 0.62, *p* = 0.032).

## Discussion

Stroke is a heterogeneous condition, making difficult the task of identifying those patients who are most likely to benefit from a given restorative therapy, and underscoring the need for predictors of individual treatment response. The current focus was on visuomotor skills, which are relevant to many activities of daily living and are often a focus of stroke rehabilitation. Here we addressed the need for clinical predictors by testing the hypothesis that, among a cohort of patients with chronic hemiparetic stroke, training-related gains in a visuomotor tracking task can be predicted by an EEG-based measure of frontoparietal circuit function that is known (13) to predict training-related gains in healthy subjects. The data support this hypothesis, finding that visuomotor tracking training significantly improves visuomotor performance after stroke, and that an EEG-based measure of frontoparietal circuit function predicts the training-related behavioral gains arising from this circuit, as hypothesized, and with specificity, i.e., behavioral gains were predicted only when examining EEG activity in the hypothesized brain circuit.

The primary study hypothesis focused on prediction using a measure of brain function, coherence in the high beta (20–30 Hz) band within a frontoparietal circuit. Measures of brain function can provide information about neurological status and its change over time beyond what can be learned from measures of brain structure or behavior, for example, providing a unique source of insights in settings ranging from genetic risk (48) to severe neural injury (49, 50). The current study focused on an EEG-based measure of brain function that was established in a prior study of healthy controls. In that study (13), brain activity was recorded in 17 healthy young subjects during 3 min of rest then examined in relation to training-related gains in right arm visuomotor tracking skill. A partial least squares regression model found that left M1 high beta band coherence, particularly with left PAR area, was a strong predictor of visuomotor skill acquisition, with most of the prediction arising from significant left M1-parietal coherence (*r* = 0.58, *p* < 0.05). In that study, these EEG-based findings exceeded the predictive value provided by baseline behavior and demographics. Based on this, coherence between leads overlying iM1 and iPAR was hypothesized to predict paretic arm training-related gains in visuomotor skill. The data support the primary study hypothesis, with a similar relationship (*r* = 0.61, *p* = 0.037) identified in the current cohort of subjects with chronic hemiparetic stroke. This finding must be interpreted in light of the fact that the results in Figure 3 are in part driven by the two patients who had the highest baseline coherence and the highest training-related behavioral gains. A weakness of the current study was the absence of a control group. An inactive control could provide insights into the main effect of time, while an active control group performing a different training task could provide insights that are more specific to visuomotor processing. Current results focused on the iM1-iPAR coherence in the high beta frequency band, but a secondary analysis found that results were also significant with delta frequency coherence. Although measures of delta band power are generally associated with neural injury, considerably less is known regarding delta band coherence, and so this finding suggests that delta range coherence may be a useful measure of motor system function worthy of further study in the context of brain plasticity.

The brain functional measure of interest was activity within a specific frontoparietal circuit. Current results reinforce that measures of circuit activity provide behaviorally relevant insights into a network's functional status in the setting of stroke (9, 51–54). EEG coherence between two brain regions likely reflects their functional connectivity, though the influence of common drive from a third brain region cannot be excluded (14, 15). Findings were specific to the hypothesized iM1-iPAR circuit (Table 3), consistent with known visuomotor functions of these brain areas in relation to the content of training (16, 20–25).

Incorporating measures of both neural function and neural injury improves prediction of stroke rehabilitation therapy effects. Anatomical details are important when evaluating physiology-behavioral relationships (9, 54), and so the relationship between iM1-iPAR coherence and training-related gains in visuomotor skill was reexamined excluding patients with injury to either iM1 or iPAR. Despite reducing sample size, removing these four patients increased the significance of the relationship between baseline EEG and training-related gains, highlighting the importance of measuring stroke-related injury to regions for which function is being assessed. Change in high beta band iM1-iPAR coherence did not correlate with training-related gains in visuomotor skill, consistent with a prior study that found that resting EEG is a better predictor than it is a biomarker of change (31). That resting EEG data predicts gains from 1 week of training but does not change in parallel with training suggests that resting EEG measures correspond to features of functional brain organization that are highly stable and do not rapidly change. This may be because the type of brain plasticity needed to change EEG coherence over time requires a large dose of training; a change in EEG coherence requires a change in two brain areas' relationship, and this may be a complex neural task. Consistent with this, in a study where subjects received 28 days, rather than 4 days, of training, we did find that measures of high beta coherence with iM1 changed in parallel with training-related behavioral gains (10). Inter-subject variability in neural plasticity over time or Type II error might have also contributed to the observed lack of association found between change in iM1-iPAR coherence and change in % SR score over time.

Increasing evidence supports the utility of using computer-based games to provide rehabilitation that improves outcomes after stroke (55–57). The current study used augmented reality, in which virtual objects are projected into the real world, to drive visuomotor skill training through 6 games (Figure 2). An augmented reality approach has several potential advantages that can enhance post-stroke therapy, for example, patients can interact with any object that can be displayed in their visual field, safely, in a game-play context. Augmented reality also has potential advantages compared to rehabilitation-focused games played on a typical computer monitor, as an augmented reality approach can decrease cognitive demands, such as a visuospatial transformation from third-person to first-person space (34). These games were implemented using home-based telehealth methods, building on an approach that we have described previously (58). The current study found significant training-related gains in a visuomotor tracking task and so highlights the utility of an augmented reality gaming approach as part of stroke rehabilitation, and provides preliminary evidence supporting home-based augmented reality gaming.

The behavior at the center of training, visuomotor tracking, was selected in part because of its clinical relevance to many functional tasks (59). One key area of focus during stroke rehabilitation involves visuomotor behaviors such as reaching and grasp, as these are essential for many activities of daily living. The current study had subjects train on a novel augmented reality gaming system that focused on gross arm movement and reaching. Guided reaching tasks have been shown to involve the posterior parietal cortex (60–62), and here we have found this region to be an important element within the frontoparietal circuit that predicted improvement in our motor tracking task. The validation of the importance of this frontoparietal circuit in visuomotor tasks could have implications for stroke rehabilitation. The current form of visuomotor training was associated with generalization, i.e., two of the three tests of visuospatial skill also improved (Table 2), although a non-treated control group would be needed to insure that this was not in part attributable to learning effects from repeated testing. Being able to target specific circuits has been shown by our group as a possibility of rehabilitation to optimize motor recovery outcomes (63, 64).

Predictors are important in designing individual rehabilitation treatment programs after stroke, providing measures that stratify patients into clinically useful categories (65, 66). The current study focused on EEG measures of functional connectivity, but other forms of connectivity such as structural connectivity may also be useful (67, 68). EEG is a useful tool, particularly for studies of the motor system (13, 31, 69). Compared to other imaging-based prediction techniques, EEG has potential advantages such as low cost, good safety, and high accessibility in complex medical settings. The current study found that an EEG-based measure, high beta band coherence within a frontoparietal circuit function at rest, predicted training-related visuospatial behavioral gains arising from that circuit, with specificity. Ultimately, such findings may lead to broader incorporation of functional brain measurements into the management of stroke rehabilitation.

## Author contributions

RZ and JC: study design, data collection, data analysis, and writing; HH, MK, LD, AM, CL, and WS: study design; KW, FE, and NK: data collection; DY: data analysis; RS and SC: study design, data analysis, and writing.

### Conflict of interest statement

The authors declare that the research was conducted in the absence of any commercial or financial relationships that could be construed as a potential conflict of interest.
